# Supplementary material for: Patterns of Intron Gain and Loss in Fungi
Source: PLoS Biol. 2004 Nov 30;2(12):e422. doi: 10.1371/journal.pbio.0020422 (PMC532390; doi:10.1371/journal.pbio.0020422)
Supplement: Table S1 — Also available at http://genes.mit.edu/NielsenEtAl/. (4.3 MB ZIP). [file pbio.0020422.st001.zip › NielsenEtAl/html/1188.html]

AN3029.1.NCU09721.1.MG03320.1.FG06317.1


```
 CLUSTAL W (1.82) Multiple Sequence Alignments - Introns Inserted


Sequence 1: NCU09721.1	749 aa
Sequence 2: MG03320.1	738 aa
Sequence 3: FG06317.1	747 aa
Sequence 4: AN3029.1	766 aa
Alignment Length: 786 aa
Number Identitical Residues: 522 aa
Alignment Score (without introns) 21419


MG03320.1 	MSAVNRIRGALAGPRKGETFELRAGLV~SQYAYER~KEAIQKTIMAMTLGKDVSALFPDV
NCU09721.1	MSAINRIRGAFAPPRKGETFELRAGLV~SQYAYER~KEAIQKTIMAMTLGKDVSALFPDV
FG06317.1 	-MAVNRIKGAFAVPRKGETFELRAGLV~SQYAYER~KESIQKTIMAMTLGKDVSALFPDV
AN3029.1  	-MAMNRIRGAFAVPRKGETFELRAGLV2SQYAYER2KEAIQKTIMAMTLGKDVSALFPDV
          	  *:***:**:* ************** ******* **:*********************

MG03320.1 	LKNIATADLDQKKLVYLYLM2NYAKSHPDLCILAVNTFVQDSEDPNPLIRALAIRTMGCI
NCU09721.1	LKNIATSDLDQKKLVYLYLM2NYAKSHPDLCILAVNTFVQDSEDPNPLIRALAIRTMGCI
FG06317.1 	LKNIATGDLDQKKLVYLYLM2NYAKSHPDLCILAVNTFVQDSEDPNPLIRALAIRTMGCI
AN3029.1  	LKNIATSDLEQKKLVYLYLM2NYAKSHPDLCILAVNTFVQDSEDPNPLIRALAIRTMGCI
          	******.**:********** ***************************************

MG03320.1 	RVDKMVDYMEEPLRKTLRDESPYVRKTAAICVAKLFDLNPTMCIENGFLETLQELIGDPN
NCU09721.1	RVDKMIDYMEEPLRKTLRDESPYVRKTAAICVAKLFDLNPTMCIENGFLETLQEMIGDPN
FG06317.1 	RVDKMVDYMEEPLRKTLRDESPYVRKTAAICVAKLFDLNPQMCIENGFLEALQELIGDPN
AN3029.1  	RVDKMIDYMEEPLRKTLRDESPYVRKTAAICVAKLFDLGPAMCLENGFLEMLQEMIGDPN
          	*****:********************************.* **:****** ***:*****

MG03320.1 	PMVVANSVQALSEIAETAPETRALVITPATLKKLLMALNECTEWGRVTILSTLADYPPSD
NCU09721.1	PMVVANSVQALSEIHETAPETRALVLTPATLKKLLMALNECTEWGRVTILTTLAEYTAHD
FG06317.1 	PMVVANSVTALSEITETAPETRALIVTPVTLKKLLMALNECTEWGRVTILTTLADYSASD
AN3029.1  	PMVVANSVTALSEIHHAAPETQALQVTPNTLRKLLMALNECTEWGRVTILTTLSEYRTSA
          	******** ***** .:****:** :** **:******************:**::* .  

MG03320.1 	VKESEHVCERVAPQFQHVNPSVVLAAVKVVFIHMKVINPDSVRQYLKKMAPPLV1TLVAS
NCU09721.1	VKESEHICERVAPQFQHVNPSVVLAAVKVVFTHMKALNPKTVSAYLKKMAPPLV~TLIAS
FG06317.1 	QKESEHICERVAPQFQHVNPSVVLAAVKVVFIHMKALNPDSVRSYLKKMAPPLV1TLVAS
AN3029.1  	VNEAEQICERVAPQFQHANPSVVLAAVKTVFLHMKIINAELSKNYLKKMAPPLV1TLVSS
          	 :*:*::**********.**********.** *** :*..    ********** **::*

MG03320.1 	APEVQYVALRNIDLLLQAKPDILSKELRVFFCKYNDPPYVKMQKLEIMVRIANDKNFEQL
NCU09721.1	APEVQYVALRNIDLLLQANPDILSKELRVFFCKYNDPPYVKLQKLEIMVRIANDKNFDQL
FG06317.1 	QPEVQYVALRNIDLLLQAKPDILSKEMRVFFCKYTDPPYVKLQKLEIMVRIANEHNYEQL
AN3029.1  	APEVQYVALRNIDLLLQKQPDILNKELRVFFCKYNDPPYVKFQKLEIMVRIANDRNFDQL
          	 **************** :****.**:*******.******:***********::*::**

MG03320.1 	LAELKEYALEVDMDFVRRAVKAIGQVAIKIESASEKCVNALLDLMATKVNYVVQEVVVVI
NCU09721.1	LSELKEYALEVDMDFVRRAVKAIGQVAIKIEAASEKCVNALLDLISTKVNYVVQEVIVVI
FG06317.1 	LAELKEYALEVDMDFVRRAVKAIGQVAIKIENAAEKCVAALEDLISTKVNYVVQEVIVVI
AN3029.1  	LAELREYALEVDMDFVRRAVKAIGQVAIKIESACEKCVNTLLDLINTKVNYVVQEAIVVI
          	*:**:************************** *.**** :* **: *********.:***

MG03320.1 	KDILRKYPGYEGVIPTLCEHIDELDEPNARGSLIWIVGEYAEKISNADEIISTFVDGFME
NCU09721.1	KDILRKYPGYEGVIPTLCKYIDELDEPNARGALIWIVGEYAEKINNADEILEGFVEGFLE
FG06317.1 	KDILRKYPGYEGVIPTLCKHIDELDEPTARGSLIWIVGEYAEKINNADEILESFVESFME
AN3029.1  	KDIFRKYPGYEGIIPTLCKCIDELDEPNARAALIWIVGEYAEKISNAGDILAGFVEGFNE
          	***:********:*****: *******.**.:************.**.:*:  **:.* *

MG03320.1 	EFT-------------------Q0TQLQILTAAVKLFLKKPSNNQGLVQKVLQQATADND
NCU09721.1	EFT-------------------Q~TQLQILTAVVKLFLKKPSNTQGLVQKVLQQATADND
FG06317.1 	EFT-------------------Q0TQLQILTAVVKLFLKKPGSNQNLVQKVLQAATAEND
AN3029.1  	EFSQVCSIHILRKPSSHINGQWQ~TQLQILTAVVKLFLKRPDKAQGLVQKVLQAATAEND
          	**:. .:     ..::  ... * ********.******:*.. *.******* ***:**

MG03320.1 	NPDIRDRAYVYWRLLS--GDLDIAK0NIITSQKPAITTTMTSLPSSLLEQLLTELSTLAS
NCU09721.1	NPDIRDRAYIYWRLLS--GDLDVAK0SIILSQKPAITTTVTSLPPVLLEQLLSELSTLAS
FG06317.1 	NPDIRDRAYVYWRLLS--SDPEVAK0NIVLSQKPTITTTMTSLPPALLEQLLTELSTLAS
AN3029.1  	NPDVRDRAYVYWRLLSNTSDPDAAR~NIVLSKKPPIVTTIHSLPPALLEQLLTELSTLAS
          	***:*****:******.:.* : *: .*: *:**.*.**: ***. ******:*******

MG03320.1 	VYHKPPESFVGKGRFGADAIQRAAIQ~EQRRDAAENPIAAAVAAAAN---GAPPQSNVEN
NCU09721.1	VYHKPPESFVGKGRFGADEIQRAAIQ~EQRQNAAENPIAASVAAAAK---GGQ-QNNVEN
FG06317.1 	VYHKPPESFVGKGRFGADEIQRAAIQ~EQRQNAAENPIAASVATASSNGSGGGAQNNIEN
AN3029.1  	VYHMPPEQFVGQGRFGADAVQKAAIE2EQLQNARENPLAAAAAAAVDGTAAPQQQNNVEN
          	*** ***.***:****** :*:***: ** ::* ***:**:.*:* .. :.   *.*:**

MG03320.1 	LLDIDFDGAAPASAE---------ASG---TPDRVAS-------PGVPAAPSGGMADMMG
NCU09721.1	LLDIDFDGGAPASAE---------HGGGVGTPDRFGSPANVGGLPGAPPQPSGGMADIMG
FG06317.1 	LLDIDFDGAAPASHE---------QNSASATPDRVAS-------PSAS-GPSGAMADMMS
AN3029.1  	LLDIDFDGTAPASAHKEPGGGVSGLDGLAGTPVRVDSP------AGGAPSGSNNLDDLLG
          	******** **** ....... :. ..  .** *. *.      .. ..  *. : *::.

MG03320.1 	LFGDMGGGSSAPAPSAAAP-SSGMDDIMGGFAGLDMGG--SSAPPPATQQLQGGAGAGAK
NCU09721.1	LFDAPPPMPSASAATSPAA-AGGMGDMMSSFAGMDLSG--TSAPPPPGQQLGHPASQTPP
FG06317.1 	MFDAPAQPSGVGAPTSAAGPSNNMNDLMNGFEGLNFGGATTSEPLPAAMQLQQAQGGAPQ
AN3029.1  	VFGDN-VQSSTGAHAPPTG--GAGADLLNGFSGLDLSG-NMSSPPPASQ---------SS
          	:*.     ... * :..:   .   *::..* *:::.* . * * *.           . 

MG03320.1 	KN--DNEDLLGLF
NCU09721.1	KSGGGSEDLLGLF
FG06317.1 	QPKKDSDDLLGLL
AN3029.1  	QPKKTNEDILSLF
          	:    .:*:*.*:
```
